# Odorous and Non-Fatal Skin Secretion of Adult Wrinkled Frog (*Rana rugosa*) Is Effective in Avoiding Predation by Snakes

**DOI:** 10.1371/journal.pone.0081280

**Published:** 2013-11-21

**Authors:** Yuri Yoshimura, Eiiti Kasuya

**Affiliations:** 1 Department of Biology, Graduate School of Systems Life Sciences, Kyushu University, Fukuoka, Japan; 2 Department of Biology, Faculty of Sciences, Kyushu University, Fukuoka, Japan; University of Lleida, Spain

## Abstract

The roles played by nonfatal secretions of adult anurans in the avoidance of predation remain unknown. The adult Wrinkled frog (*Rana rugosa*) has warty skin with the odorous mucus secretion that is not fatal to the snake *Elaphe quadrivirgata*. We fed *R. rugosa* or *Fejervarya limnocharis*, which resembles *R. rugosa* in appearance and has mucus secretion, to snakes and compared the snakes’ responses to the frogs. Compared to *F. limnocharis*, *R. rugosa* was less frequently bitten or swallowed by snakes. The snakes that bit *R. rugosa* spat out the frogs and showed mouth opening (gaping) behavior, while the snakes that bit *F. limnocharis* did not show gaping behavior. We also compared the responses of the snakes to *R. rugosa* and *F. limnocharis* secretions. We coated palatable *R. japonica* with secretions from *R. rugosa* or *F. limnocharis*. The frogs coated by *R. rugosa* secretion were less frequently bitten or swallowed than those coated by *F. limnocharis* secretion. We concluded that compared to different frog species of similar sizes, the adult *R. rugosa* was less frequently preyed upon by, and that its skin secretion was effective in avoiding predation by snakes.

## Introduction

Many animals attack and prey upon adult anurans [1,2]. Adult anurans have evolved various defense mechanisms including chemical ones [2]. Anuran chemical defenses against predators have been studied extensively in poison frogs, which include some species of *Dendrobates* and Bufonidae with extremely poisonous skin secretions [2]. These studies have shown that the skin secretions of adult poison frogs are toxic enough to kill their predators immediately [3].

In addition to such extremely poisonous species, most adult anurans have highly glandular skin that emits secretions [2,4]; these skin secretions often provide antibacterial protection [2,4–8]. The secretions of adult anurans other than the extremely poisonous species are not considered to be fatal, but are suggested to repel predators by irritating the mucus membranes of the mouth and causing regurgitation, impairing coordination, or affecting their chemical senses [2,4,9]. For example, the secretion of the African clawed frogs (*Xenopus laevis*) induce dyskinetic orofacial behavior (yawning and gaping movements) in the northern water snake *Nerodia sipedon* [10] and in two other snake species (*Lycodonomorphus rufulus* and *L. laevissimus*)[11].

The role that such nonfatal secretions play in the avoidance of predation remains unknown. Although the extremely poisonous secretions cause serious illness or death in predators [2,9], animals with poisonous secretions can avoid predation before contact with predators and are seldom actually killed because many predators learn to avoid or exhibit innate avoidance of these species [2]. Nonfatal secretions can be effective after contact with predators or they invoke avoidance responses before contact as in the case of the extremely poisonous ones. Because contact and handling by predators negatively affect the prey [9], it is important to understand whether predation is avoided before or after contact to assess the effectiveness of secretions as a defense mechanism. However, it is unclear how nonfatal secretions prevent predation. Only the studies by Barthalmus and Zielinski [10,11], who reported that the *X. laevis* secretion is effective in anti-predator defense by inducing dyskinetic orofacial behavior (including gaping, yawning, and writhing tongue) in *N. sipedon*, have shown a behavioral mechanism for predation avoidance by nonfatal secretions. These researchers showed that the frog was able to escape from the snake during the dyskinetic orofacial behavior after contact with the secretion. However, this behavior may be of limited effectiveness in predation avoidance because dyskinetic orofacial behavior is ineffective for escape from the snakes that coil around prey to hold them (e.g., *Lycodonomorphus* snakes).

The adult Wrinkled frog (*Rana rugosa*) has warty skin with a secretion that has a strong and unique odor. This frog is rarely found in the natural diet of the Japanese striped snake (*Elaphe quadrivirgata*), which is considered a general predator of amphibians, mammals, birds, and reptiles [12]. In one study, newborn *E. quadrivirgata* with no prey experience ate few *R. rugosa* [13]. When *E. quadrivirgata* adults were forced to swallow *R. rugosa*, all the snakes spat out the frogs and opened and closed their mouths (gaping behavior) (Yoshimura, personal observation). The snake did not change its movements or other behaviors and did not die shortly after contact with *R. rugosa* (Yoshimura, personal observation). These observations suggest that *R. rugosa* are not highly poisonous but that they escape from predation by snakes. The absence of species with highly poisonous adults in the subgenus *Lithobates*, which includes *R. rugosa* [2], also suggests that *R. rugosa* is not highly poisonous.

We conducted two experiments to examine whether the skin secretion of adult *R. rugosa* is effective for the evasion of predation by snakes. In the first experiment (Experiment 1), we compared the proportion of snakes that bit and swallowed *R. rugosa* with the proportion that bit and swallowed *Fejervarya limnocharis*, which resembles *R. rugosa* in size and appearance. In the second experiment (Experiment 2), we coated the natural prey organisms of the snakes with secretions from *R. rugosa* or *F. limnocharis* to examine the effects of these secretions.

## Materials and Methods

### Ethics statement

The study was conducted in accordance with Act on Welfare and Management of Animals (Law No. 105, Japan). All experiments followed the ABS/ASAB guidelines for ethical treatment of animals. The Animal Care and Use Committee of Kyushu University approved this study. All collection in this study was carried out on private lands, and we confirm that the owners of the lands gave permission to collect these animals. This study did not involve any endangered or protected species.

### Predators: Snakes

We used 34 adult Japanese striped snakes (*E. quadrivirgata*; mean =1086 mm, SD=142) collected in Fuji, Saga Prefecture, Japan (33°24′01″N, 130°09′39″E), in June and July 2009, as the predators in the experiments. This species was considered the most abundant among potential predators of frogs at the site because we encountered them most frequently among potential predators (Yoshimura, personal observation). Snakes were collected by hand, and transferred to the laboratory in clean cloth bags with temperature maintained at 23–28°C to reduce the load of them. Upon collection, we stretched each snake along a tape measure and recorded its total body length (snout–vent length and tail length) to the nearest 5 mm. The snakes were housed individually in polypropylene containers (450 × 295 mm and 260 mm high) prior to the experiments, and were subjected to a 12 h L:12 h D photoperiod with temperature maintained at 24–25°C. Water was provided in saucers (180 mm diameter and 50 mm deep), and the snakes were able to drink and bathe at any time during housing. Each snake was fed one Japanese brown frog (*R. japonica*) or one Japanese meadow frog (*R. nigromaculata*) every 2 days.

### Prey: Frogs

We used adult *R. rugosa* (28–47 mm), Indian rice frog (*F. limnocharis*, 25–46 mm), and Japanese brown frog (*R. japonica*, 28–32 mm) as the prey items in the experiments. *Rana rugosa* and *R. japonica* were collected from the same area that the snakes were collected, in July 2009. Because *F. limnocharis* was not found in the area, we collected this species at Motooka, Fukuoka Prefecture, Japan (33°35′48″N, 130°12′52″E), in July 2009. *Fejervarya limnocharis* and *R. japonica* had odorless skin secretions for human. The *E. quadrivirgata* that we used in this study are likely to encounter *R. rugosa* and *R. japonica*, but not *F. limnocharis*, in this sampling site (Yoshimura, personal observation). All frogs were caught with a landing net, and transferred to the laboratory in plastic cages with water temperature maintained at 23–28°C to reduce the load of them. We measured the snout–vent length of all frogs to the nearest 0.1 mm by using a vernier caliper.

After collection, five frogs were maintained in one polypropylene aquarium (600 × 300 mm and 200 mm high) for 24–36 h prior to the experiments. They were subjected to the natural photoperiod, and temperature was maintained at 20–25°C. The water depth in the aquarium was approximately 20 mm. Each aquarium had a land (100 × 150 mm) on one side. The frog aquaria and snake containers were placed in different rooms to avoid detection of scents and visual stimuli between the frogs and snakes before the experiments.

### Experimental design

We conducted Experiments 1 and 2. In Experiment 1, we fed the snakes either *R. rugosa* or *F. limnocharis* and compared the proportion of snakes that bit or swallowed each species. In Experiment 2, we fed the snakes *R. japonica* coated with the secretions of either *R. rugosa* or *F. limnocharis*, and compared the proportion that bit or swallowed *R. japonica* coated with the two species’ secretions. To test the effect of their secretions, we used *R. japonica*, which were eaten by the snakes and had similar body size as that of *R. rugosa* and *F. limnocharis* (approximately 30 mm), as the prey. We also compared the time from the first biting to the second biting (biting interval) in Experiment 2. We used each frog in only one of the experiments.

Before the experiments, the snakes had been trained to eat frogs in the aquaria where the experiments were conducted. Three days before the experiments, from 9:00 AM to 4:00 PM, we put one snake in each experimental aquarium for 5 min for acclimation. We then put one *R. japonica* in each aquarium and observed the aquaria by using video cameras (DCR-SR87, Sony, Japan) for 20 min after the introduction of the frogs. The snakes usually fed within 20 min. We used 34 snakes that bit the frogs during this training, while the five snakes that did not bite the frogs were excluded. The snakes were individually housed without food for 24 h before the experiment.

The training and the two experiments were conducted in glass aquaria (600 × 300 mm and 450 mm high) with the temperature of the room maintained at 24–25°C. We covered the tops of the aquaria with white polypropylene boards (600 × 300 mm) to prevent the snakes from escaping. We covered three of the four sides of each aquarium with gray boards to block other snakes and frogs from view during the training and experiments. We observed the snakes using video cameras (DCR-SR87, Sony, Japan) from the sides of the aquaria that were not covered by the boards.

### Experiment 1

In Experiment 1, each snake was tested using the frogs two times. A snake was given *R. rugosa* on one day and *F. limnocharis* on the other day. We chose to use *F. limnocharis* as the control to minimize the differences in visual appearance with *R. rugosa*, as *F. limnocharis* resembled *R. rugosa* in color, size, and the presence of small ridges on its back [14,15]. *Fejervarya limnocharis* was usually included in the diet of *E. quadrivirgata* in the area where *F. limnocharis* was distributed [12]. We used 34 snakes in this experiment, randomly divided into two groups of 17 snakes. Seventeen of the 34 snakes were fed *R. rugosa* on the first day and *F. limnocharis* on the second day, while the other 17 snakes were fed *F. limnocharis* on the first day and *R. rugosa* on the second day. We used each frog in only one of the experiments.

On the first day of Experiment 1, after the 48–54 h no-feeding period, we put one snake in each aquarium for 5 min for acclimation. After the 5-min acclimation period, we fed the snake one frog and observed the animals by using video cameras for 20 min. We returned the snakes to their housing containers after the first day of experiments and did not provide food to the snakes until the test on the next day (the second day). On the second day, we tested the snakes with the other species of frog in the same manner.

### Experiment 2

We compared the responses of the snakes to secretions of *R. rugosa* and *F. limnocharis*. We coated *R. japonica* with secretions from *R. rugosa* (termed “wrinkled frog”) or *F. limnocharis* (control) and again tested each snake twice. Snakes were fed a “wrinkled frog” on one day and the control on the other day.

Coating of *R. japonica* with the *R. rugosa* secretion was performed as follows. First, we wiped the body surfaces of all the frogs with paper towels to remove extra water. We picked at the right or left half of the back skin of one *R. rugosa* 10 times slowly with tweezers (INOX50) to allow the secretion to ooze out onto all the surface including the limbs. To apply the secretion, we rubbed the half of the *R. rugosa* back skin, which had the secretion oozing out, directly against the entire body surface of the *R. japonica* (including the ventral side and back). For the control, we coated *R. japonica* with the secretion from *F. limnocharis*. The procedures for the control were the same as those for the “wrinkled frog” except for the different frog species used as the source of the secretion. We used each frog in only one of the experiments. Three *R. japonica* lost their activity after the coating; we did not use these frogs because inactive preys could affect the predatory behavior of the snakes.

Among the snakes used in Experiment 1, 16 randomly chosen snakes were used in Experiment 2. These snakes were kept for 21–28 d after Experiment 1 under housing conditions, and were randomly divided into two groups of eight snakes. Eight of the 16 snakes were given the “wrinkled frog” on the first day and the control on the second day; the other eight snakes were given the control on the first day and the “wrinkled frog” on the second day.

On the first day of Experiment 2, after the 48–54 h no-feeding period, we put one snake in each aquarium for acclimation. After the 5-min acclimation period, we put one “wrinkled frog” or one control in each aquarium and observed the animals with the video camera for 20 min. We returned the snakes to their housing containers after the first day of experiments and did not provide food between the first and second day of this experiment. On the second day, we tested the snake with the other type of the frog in the same manner.

Because we improved the placement of the video cameras in Experiment 2, we were able to precisely record the movement of the mouths of the snakes holding the frogs than in Experiment 1. After the snakes bit the frogs (first biting), they held the frogs in their mouths and bit them again (second biting) and then swallowed them. The snakes held the frogs in their mouths during the interval between the first and second biting (biting interval). When the snakes were fed *R. rugosa*, those that bit but did not swallow the frogs released the frogs in the biting interval without making a second bite. We considered this interval as the time needed for the snakes to decide whether to swallow the frogs based on stimuli, including taste. In Experiment 2, we compared the biting intervals of the snakes between the “wrinkled frog” and the control. The biting interval will be long if the secretion creates a strong disturbance in swallowing the prey. We measured the biting interval to the nearest 1 s.

### Statistical analysis

We used Fisher's exact test to compare the proportions of the individuals that showed biting, swallowing, or gaping behaviour. For the biting interval, we used Wilcoxon signed rank test. The Wilcoxon test was used to compare the paired data of the interval that a given snake took for the “wrinkled frog” and that the snake took for the control. Statistical significance was designated for differences with p-values less than 0.05. We performed all the analyses in R 2.15.1 [16].

## Results

### Experiment 1: Predation on *R. rugosa* and F. *Limnocharis*

Table 1 shows the number of snakes that bit or swallowed *R. rugosa* or *F. limnocharis*. The proportion of snakes that bit *R. rugosa* was 17.6% (3/17) on the first and second days, while the proportion of snakes that bit *F. limnocharis* was 82.4% (14/17) on the first day and 58.8% (10/17) on the second day. A significantly lower proportion of snakes bit *R. rugosa* (17.6% = 6/34) than *F. limnocharis* (70.6% = 24/34) (Fisher’s exact test; p = 0.0071).

**Table 1:** Number of snakes that bit or swallowed *R. rugosa* or *F. limnocharis*, and gaping behavior.

	Bit		Swallowed		Gaping behavior	
	*R. rugosa* (%)	*F. limnocharis* (%)	*R. rugosa* (%)	*F. limnocharis* (%)	*R. rugosa* (%)	*F. limnocharis* (%)
First day	3/17 (17.6)	14/17 (82.4)	0/17 (0)	14/17 (82.4)	3/3 (100)	0/14 (0)
Second day	3/17 (17.6)	10/17 (58.8)	0/17 (0)	10/17 (58.8)	3/3 (100)	0/10 (0)
Total	6/34 (17.6)	24/34 (70.6)	0/34 (0)	24/34 (70.6)	6/6 (100)	0/24 (0)

No snakes swallowed *R. rugosa* on the first or second day (0/17 on each day). The proportion of snakes that swallowed *F. limnocharis* was 82.4% (14/17) on the first day and 58.8% (10/17) on the second day. A significantly lower proportion of snakes swallowed *R. rugosa* (0% = 0/34) than *F. limnocharis* (70.6% = 24/34) (Fisher’s exact test; p = 0.000027). The proportion of snakes that swallowed *R. rugosa* to snakes that bit *R. rugosa* (0% = 0/6) was significantly lower than that which bit *F. limnocharis* (100% = 24/24) (Fisher’s exact test; p = 0.028).

A significantly higher proportion of snakes showed gaping behavior after biting *R. rugosa* (100% = 6/6) than *F. limnocharis* (0% = 0/24) (Fisher’s exact test; p = 0.00047).

### Experiment 2: The effect of the secretion of *R. rugosa*

Table 2 shows the number of snakes that bit or swallowed the “wrinkled frog” or the control. The proportion of snakes that bit the “wrinkled frog” was 62.5% (5/8) on the first day and 75.0% (6/8) on the second day. The proportion of snakes that bit the control was 100% on the first and second days (8/8 on each day). A significantly lower proportion of snakes bit the “wrinkled frog” (68.8% = 11/16) than the control (100% = 16/16) (Fisher’s exact test; p = 0.0043).

**Table 2:** The number of snakes that bit or swallowed “wrinkled frog” or the control.

	Bit		Swallowed	
	“Wrinkled frog” (%)	Control (%)	“Wrinkled frog” (%)	Control (%)
First day	5/8 (62.5)	8/8 (100)	4/8 (50.0)	8/8 (100)
Second day	6/8 (75.0)	8/8 (100)	5/8 (62.5)	8/8 (100)
Total	11/16 (68.8)	16/16 (100)	9/16 (56.3)	16/16 (100)

The proportion of snakes that swallowed the “wrinkled frog” was 50% (4/8) on the first day and 62.5% (5/8) on the second day, while the proportion of snakes that swallowed the control was 100% on the first and second days (8/8 on each day). A significantly lower proportion of snakes swallowed the “wrinkled frog” (56.3% = 9/16) than the control (100% = 16/16) (Fisher’s exact test; p = 0.0068). The proportion of snakes that swallowed the “wrinkled frog” to the snakes that bit them (81.8% = 9/11) was lower than that which swallowed the control to the snakes that bit them (100% = 16/16), but the difference was not significant (Fisher’s exact test; p = 0.28).

The proportion of snakes that bit the “wrinkled frog” among those that showed gaping (72.7% = 8/11) was significantly higher than the proportion of snakes that bit the control among those that showed gaping (0% = 0/16) (Fisher’s exact test; p = 0.0038). One snake that released the “wrinkled frog” on the first day showed gaping while one snake that released the “wrinkled frog” on the second day did not.

When we fed the “wrinkled frog” on the first day, four out of the eight snakes bit it twice and the average biting interval was 91.5 s (SD = 37.8). On the second day, the average biting interval for five of the eight snakes was 149.4 s (SD = 67.5). When we gave the control on the first day, the average biting interval for the eight snakes was 28.9 s (SD =17.5). On the second day, the average biting interval for the eight of the snakes was 31.5 s (SD = 11.2). In the nine snakes that bit both the “wrinkled frog” and the control, the biting interval for the “wrinkled frog” (mean = 123.7 s, SD = 61.2) was significantly longer than the biting interval for the control (mean = 30.2 s, SD = 14.3) (Wilcoxon signed rank test; V = 0, p = 0.009).

## Discussion

A significantly lower proportion of snakes bit or swallowed *R. rugosa* than *F. limnocharis* (Experiment 1), indicating that adult *R. rugosa* was less frequently preyed upon than a different frog species of similar size. No *R. rugosa* were swallowed and all survived after being bitten by snakes, while 70.6% of *F. limnocharis* were killed. In Experiment 2, a significantly lower proportion of snakes bit or swallowed the “wrinkled frog” coated with the secretion of *R. rugosa* than the control; therefore, the secretion is effective in avoiding predation by the snake.

Although some have skin secretions that are capable killing predators, most anurans are noxious to predators by irritating the mucus membranes of the mouth, causing regurgitation, or affecting their chemical senses rather than by killing them [2,4,9]. However, only Barthalmus and Zielinski [11] reported the effectiveness of such nonfatal secretions in avoiding predation; these researchers showed that the frog was able to escape from the snake during dyskinetic orofacial behavior caused by the secretion.

The roles of nonfatal secretions in avoiding predation remain unknown. In the present results, *R. rugosa* avoided predation both before and during contact with the snake. First, the difference before contact of the snake and frog can be evaluated by comparing the proportion of snakes that bit the frogs. This proportion for *R. rugosa* was lower than that for *F. limnocharis* (Experiment 1). This was also the case for the “wrinkled frog” in Experiment 2. Second, the difference during contact of the snake and frog (when the frog was in the mouth of the snake) can be evaluated by comparing of the proportion of snakes that swallowed the frogs to the number of the snakes that bit frogs. In Experiment 2, the difference during the contact can be also evaluated by the biting interval.

The difference in the proportion of biting shows that the snakes were able to recognize differences in *R. rugosa* and *F. limnocharis* before contact, and that the snakes were also able to recognize differences in the secretions (the “wrinkled frog” and control in Experiment 2). This suggests that the snakes used cues other than contact chemicals and mechanical recognition. Because the “wrinkled frog” and the control in Experiment 2 were similar in visual appearance, and hence, visual cues were not likely to be responsible for the difference. The use of olfactory cues is more likely because snakes are generally known to use olfactory recognition [17]. The snakes we used showed tongue-flick behavior (Yoshimura, personal observation), which indicates that the snakes snared chemical particles in the air [9]. Olfactory recognition by the snakes is also supported by the strong and unique smell of *R. rugosa* [15], which emanates from its skin secretion (Yoshimura, personal observation).

Our results also showed differences during contact. The proportion of snakes that swallowed *R. rugosa* to those that bit *R. rugosa* was lower than the proportions for *F. limnocharis* (Experiment 1). This proportion for the “wrinkled frog” was lower than that of the control, but the difference was not significant (Experiment 2). However, the significantly longer biting interval in the “wrinkled frog” compared to the control illustrates the difference. Although the possibility of olfactory cues (by volatiles) is not excluded in the present results, contact chemicals are the most likely factors responsible for the differences during contact. We expect that some bitter-tasting chemicals in the secretion of *R. rugosa* are responsible for the difference.

Highly poisonous anuran species can avoid predation before contact rather than killing predators after contact, because predators learn to avoid or exhibit innate avoidance of these species [2]. In our results, *R. rugosa* and its nonfatal secretion are also effective in avoiding predation by the lower frequency of contacts. However, it is unclear whether the lower frequency of contacts depends on learning or on innate abilities of the snakes. Future studies are necessary to clarify this point as that done for poisonous species.

All the snakes that bit *R. rugosa* and a majority of snakes that swallowed the “wrinkled frog” showed gaping behavior. The size of the frogs should not have affected gaping, because all of the frogs were approximately the same size. Barthalmus and Zielinski [11] suggested that yawning and gaping induced by the secretion of *X. laevis* in *N. sipedon* facilitates the frog’s escape or slows its ingestion. However, in our experiments, the snakes showed gaping after releasing *R. rugosa* or swallowing the “wrinkled frog.” Therefore, contrary to the findings by Barthalmus & Zielinski [11], we suggest that the frog does not avoid predation specifically by gaping, but that both the gaping and the lower probability of predation are consequences of the unpalatability of *R. rugosa.*

Our results for Experiment 1, which showed that a lower proportion of snakes bit and swallowed *R. rugosa* than the control, were qualitatively similar to the results in Experiment 2, but showed quantitative differences. A lower proportion of snakes bit each frog in Experiment 1 (17.6% for *R. rugosa*, 70.6% for *F. limnocharis*) than in Experiment 2 (68.8% for the “wrinkled frog”, 100% for the control). Of the proportion of snakes that swallowed the “wrinkled frog” among the snakes that bit this frog, that in Experiment 1 (0% for *R. rugosa*) was lower than in Experiment 2 (81.8% for the “wrinkled frog”). In other words, live *R. rugosa* avoided predation more effectively than a different species of frog coated with *R. rugosa* secretion. We considered several reasons for the differences between Experiments 1 and 2. First, the secretion that we used for the coating may have been insufficient in volume for some of the frogs. Second, the snakes may have recognized the taste and smell of both *R. japonica* and *R. rugosa*, thus recognizing the “wrinkled frog” as palatable prey and attempting to swallow it. In the future, we need to identify and quantify constituents in the secretion that is effective in avoiding predation. Third, *R. rugosa* may have defensive mechanisms other than its secretion.

We hypothesized that the odor of the *R. rugosa* secretion acts as a signal that lets predators avoid prey before contact (aposematism, aposematic coloration, warning displays; [18,19]). The effectiveness of warning odors in olfactory recognition by predators has also been reported in animals other than amphibians [20–26]. Terrick et al. [26] reported that garter snakes recognized and learned chemosensory characteristics to avoid noxious prey that induce illness. If the smell of *R. rugosa* can be recognized and learned by the snakes, then *R. rugosa* could avoid predation by snakes that have such chemosensory recognition.

Why did snakes avoid preying on *R. rugosa*, a species that is not fatal to them? In general, many toxic prey species have evolved bitter-tasting chemicals [27–29]. Distasteful (e.g., bitter) chemicals facilitate recognition in predators that such toxic prey is not suitable as food [29–34]. Once the bitter taste receptors have evolved to discriminate toxic food, the prey that secretes nontoxic chemicals but stimulate the predators’ bitter-taste receptors to elicit rejection response, could escape from predation [35]. The present results, in which snakes that bit *R. rugosa*, released them, and showed gaping, suggest that *R. rugosa* is unpalatable to the snakes. At present, it is not clear whether the secretion of *R. rugosa* is bitter to the snakes. It is possible that the secretion of *R. rugosa*, which is not fatal to the snake, stimulated the predators’ bitter-taste receptors, which had evolved in response to toxic and bitter-tasting items.
